# Cloning and Molecular Characterization of *CmOxdc3* Coding for Oxalate Decarboxylase in the Mycoparasite *Coniothyrium minitans*

**DOI:** 10.3390/jof8121304

**Published:** 2022-12-16

**Authors:** Yuping Xu, Mingde Wu, Jing Zhang, Guoqing Li, Long Yang

**Affiliations:** State Key Laboratory of Agricultural Microbiology and Hubei Key Laboratory of Plant Pathology, Huazhong Agricultural University, Wuhan 430070, China

**Keywords:** *Coniothyrium minitans*, CmOxdc3, oxalate decarboxylase, *Sclerotinia sclerotiorum*, oxalic acid

## Abstract

*Coniothyrium minitans* (*Cm*) is a mycoparasitic fungus of *Sclerotinia sclerotiorum* (*Ss*), the causal agent of Sclerotinia stem rot of oilseed rape. *Ss* can produce oxalic acid (OA) as a phytotoxin, whereas *Cm* can degrade OA, thereby nullifying the toxic effect of OA. Two oxalate decarboxylase (OxDC)-coding genes, *CmOxdc1* and *CmOxdc2*, were cloned, and only *CmOxdc1* was found to be partially responsible for OA degradation, implying that other OA-degrading genes may exist in *Cm*. This study cloned a novel OxDC gene (*CmOxdc3*) in *Cm* and its OA-degrading function was characterized by disruption and complementation of *CmOxdc3*. Sequence analysis indicated that, unlike *CmOxdc1*, *CmOxdc3* does not have the signal peptide sequence, implying that CmOxDC3 may have no secretory capability. Quantitative RT-PCR showed that *CmOxdc3* was up-regulated in the presence of OA, malonic acid and hydrochloric acid. Deletion of *CmOxdc3* resulted in reduced capability to parasitize sclerotia of *Ss*. The polypeptide (CmOxDC3) encoded by *CmOxdc3* was localized in cytoplasm and gathered in vacuoles in response to the extracellular OA. Taken together, our results demonstrated that *CmOxdc3* is a novel gene responsible for OA degradation, which may work in a synergistic manner with *CmOxdc1*.

## 1. Introduction

Oxalic acid (OA) is a natural organic acid with a low molecular weight. It is characterized by strong acidity, reducibility and calcium-chelating capability [1]. OA can affect human health, causing urolithiasis, hyperoxaluria, renal failure and other diseases [2]. Furthermore, OA is a typical anti-nutrient, it can reduce solubility and utilization of minerals and trace elements, such as Ca^2+^, resulting in nutritional imbalance [3]. Many plants, including purslane, spinach and tea, as well as many fungi, such as *Sclerotinia sclerotiorum*, *Aspergillus niger* and *Ceriporiopsis subvermispora* can produce OA [4,5,6,7].

OA secretion by fungi produces many benefits for mycelial growth and substrate (niche) colonization. It is well-recognized that plant pathogenic fungi utilize OA as a virulence factor or a non-specific phytotoxin in interaction with plants with the following mechanisms [8,9,10]: (*i*) acidification of plant tissues by OA, thereby improving activity of the cell wall-degrading enzymes; (*ii*) disequilibration of the redox balance in plant tissues due to the strong reducibility of OA; (*iii*) stabilization of Ca^2+^ by chelation, thereby weakening plant resistance; and (*iv*) timely inhibition of autophagy and apoptosis. In a word, OA is the key factor regulating pathogenicity of many fungal pathogens, including *S. sclerotiorum*.

Oxalate decarboxylase (EC 4.1.1.2, OxDC) is an enzyme that can degrade OA to generate formic acid and CO_2_. It has been found to exist in numerous microorganisms [6,11,12,13,14,15,16,17,18,19,20]. Most microorganisms have more than one OxDC gene. For example, there are seven OxDC genes in *Phanerochaete chrysosporium* [15]. The transcript patterns and the subcellular localization of the OxDC genes vary greatly in different microorganisms, implying that the fungal OxDCs may have diversified physiological functions. Up to now, at least three kinds of transcript patterns of the OxDC genes have been reported in fungi, including: (*i*) the plant tissue-specific expression pattern for *Ss*-*odc1* and *Ss*-*odc2* in *S. sclerotiorum*, which were not induced by low pH or exogenous OA [11]; (*ii*) OA- and HCl-triggered expression pattern in *F. velutipes* with the aid of a low-pH-responsive element (LPRE), a 13-bp sequence (5′-GCGGGGTCGCCGA-3′) in the promoter region of the OxDC gene [21]. Moreover, this expression pattern was later confirmed with the evidence that a calmodulin (CaM)-like protein FvCaMLP could specifically bind to the two non-canonical E-box elements (5′-AACGTG-3′) in the promoter region of the OxDC gene [17]; (*iii*) OA-alone-triggered expression pattern in *Postia placenta* [22]. Many studies have shown that OxDC exists both intra- and extracellularly; it is usually located in vesicles close to the plasma membrane [23,24]. The exception is the OxDC in *P. chrysosporium*, which was only detected outside the hyphal cells [25,26]. Many fungal OxDCs contain signal peptides, which can facilitate secretion of the OxDCs to the extracellular matrix [16].

Sclerotinia stem rot (SSR) caused by *S. sclerotiorum* is a notorious disease, which usually causes huge economic losses for industries of oil crops and vegetables [27]. *C. minitans* is a mycoparasite and an important biocontrol agent of *S. sclerotiorum* [28]. *C. minitans* has been registered as a commercial biocontrol agent in the United States, European Union and China [29,30,31,32]. The modes of action of *C. minitans* include mycoparasitism on hyphae and sclerotia of *S. sclerotiorum*, antibiosis through production of antifungal and antibacterial substances, as well as degradation of OA [31,33,34,35].

Regarding OA degradation, our previous study showed that in the process of interaction between *S. sclerotiorum* and *C. minitans*, OA secreted by *S. sclerotiorum* could induce the production of OxDC by *C. minitans*. After degradation of OA, the ambient pH increased to the weakly acidic and neutral levels, which significantly up-regulated expression of mycoparasitism-related genes, such as those for chitinase, glucanase and protease, for the degradation of the cell wall of *S. sclerotiorum*. These results suggest that OA degradation plays a crucial role in the interaction between *C. minitans* and *S. sclerotiorum* [16,35,36,37].

Previous studies have identified two oxalate decarboxylase genes in *C. minitans*; namely, *CmOxdc1* (GenBank Acc. No. JF718548) and *CmOxdc2* (GenBank Acc. No. JF718549) [16]. While deletion of *CmOxdc2* had no effect on OA degradation, deletion of *CmOxdc1* resulted in 40% OA degradation in an OA-containing medium [16]. Therefore, there may exist other OxDCs responsible for degradation of the remaining OA in *C. minitans*. We herein report a novel oxalate decarboxylase gene (*CmOxdc3*), as well as its sequence structure, expression pattern, subcellular localization and function.

## 2. Materials and Methods

### 2.1. Microbial Strains, Cultural Media and Cultural Conditions

Five microbial strains were used in this study, including *C. minitans* ZS-1 (CCAM Acc. No. 041057) [38], *C. minitans* ZS-1-E1 (a eGFP control strain, unpublished data), *S. sclerotiorum* 1980, *Saccharomyces cerevisiae* YTK12 and *Escherichia coli* DH5α. The cultural media included Luria-Bertani (LB) medium, potato dextrose agar (PDA), potato dextrose broth (PDB), yeast peptone dextrose agar (YPDA), yeast peptone raffinose antimycin A medium (YPRAA) and TB3 medium. LB contained (in 1000 mL water) 10 g tryptone, 5 g yeast extract and 10 g NaCl (pH 7.0), it was used to incubate *E. coli* DH5α (37 °C). PDA and PDB were prepared with fresh potato tubers, they were used to incubate *C. minitans* and *S. sclerotiorum* (20 °C). YPDA contained (in 1000 mL water) 10 g yeast extract, 20 g peptone, 20 g D-glucose and 15 g agar, it was used to incubate the yeast strain YTK12 (30 °C). YPRAA contained (in 1000 mL water) 10 g yeast extract, 20 g peptone, 20 g raffinose, and 20 μg/mL antimycin A). TB3 medium contained (in 1000 mL water) 250 g sucrose, 5 g yeast extract, 10 g casein hydrolysate, and 8 g agar, and it was used for the regeneration of protoplasts of *C. minitans* (20 °C).

### 2.2. Gene Cloning and Analysis

The genomic DNA (gDNA) and complementary DNA (cDNA) of *C. minitans* were used as templates to amplify *CmOxdc3* as well as *CmOxdc1* with the specific primers (Table S1) designed based on the genome of *C. minitans* [39,40]. Amino acid sequences encoded by *CmOxdc3* and other four OxDC genes (*CmOxdc1*, *CmOxdc2*, *FvOxdc*, *BsOxdc*) were aligned using MEGA 7.0.26 [16,17,41]. The amino acid sequences encoded by 39 OxDC genes from eighteen fungi were used to construct a phylogenetic tree using the neighbor-joining method in MEGA 7.0.26, and a bootstrap test was carried out with 1000 replicates (Table S2). Prediction of the conserved domains and the signal peptides in *CmOxdc3* and other OxDC genes were conducted using SMART (Simple Modular Architecture Research Tool) and SignalP 5.0, respectively [42,43].

### 2.3. DNA Extraction and Southern Blotting

Conidial suspension (1 × 10^7^ conidia mL^−1^) of *C. minitans* was inoculated on autoclaved cellophane membranes placed on PDA and the cultures were incubated at 20 °C in dark for 3 d. The mycelial mass was harvested for extraction of gDNA using the CTAB method (CTAB = cetyltrimethylammonium bromide) [44]. Southern blot analysis was done to confirm disruption and complementation of genes in mutants of *C. minitans* using the Gene Images AlkPhos Direct Labeling and Detection System from GE Healthcare (Amersham Biosciences, Buckinghamshire, UK). The gDNA of the wild type was completely digested with *Nc*o I, and the gDNAs of the disruption and complementation mutants were completely digested with *Kp*n I and *Bam*H I. The DNA sequence at the left flank of *CmOxdc3* was obtained by PCR and labeled as the probe.

### 2.4. RNA Extraction and Quantification of Gene Expression

Total RNA was extracted from the mycelial masses of the wild type and mutants of *C. minitans* using E.Z.N.A. Fungal RNA Kit (TaKaRa, Dalian, China) following the manufacturer’s instructions. PrimeScript^TM^ RT reagent Kit with gDNA Eraser (TaKaRa) was used to synthesize cDNA, and TB Green^TM^ Premix Ex Taq^TM^ II (TaKaRa) was used for fluorescence quantitative PCR (RT-qPCR) with the primers listed in Table S1. The actin gene (*Cmactin*) was chosen as internal control [16]. The relative expression level of each gene was calculated by using the 2^−ΔΔCt^ method [45].

### 2.5. Gene Expression Analysis of CmOxdc3

The expression pattern of *CmOxdc3*, as well as *CmOxdc1* in the wild type (ZS-1), was detected by RT-qPCR with the RNA from the mycelial masses in the following assays: (*i*) the OA assay, 3-day-old cultures (150 rpm, 20 °C) of ZS-1 in PDB followed with 1-h treatment by amended with OA at 0 (control), 12, 24 or 32 mM; (*ii*) the acid assay, 3-day-old PDB cultures of ZS-1 (150 rpm, 20 °C) followed with 1-h treatment under pH 3 with OA (12 mM), acetic acid (75 mM), citric acid (50 mM), fumaric acid (50 mM), HCl (6 mM), lactic acid (50 mM), maleic acid (10 mM) or malonic acid (20 mM); and (*iii*) the pH assay, 2-h treatment of the 3-day-old PDA cultures of ZS-1 on cellophane films (20 °C) under pH 3, 4, 5 or 6 adjusted with 0.1 M citric acid-sodium phosphate buffer, the mycelial mass without extra pH treatment was used as control. There were three replicates for each treatment, and the entire experiment was repeated three times.

### 2.6. Gene Knockout and Complementation

*CmOxdc3*, as well as *CmOxdc1*, were separately disrupted using the split marker system [46] as shown in Figure S1A. For disruption of *CmOxdc3*, the 5′-flank region (772 bp) and the 3′-flank region (810 bp) of *CmOxdc3* were cloned and fused with the partial neomycin resistance gene (*NEO*). The two fused fragments, *CmOxdc3*-5′-NE (1614 bp) and EO-*CmOxdc3*-3′ (1816 bp), were then simultaneously transformed into the protoplasts of ZS-1. For disruption of *CmOxdc1*, the 5′-flank region (875 bp) and the 3′-flank region (843 bp) of *CmOxdc1* were cloned and fused with the partial hygromycin resistance gene (*HYG*). The two fused fragments, *CmOxdc1*-5′-HY (1949 bp) and YG-*CmOxdc1*-3′ (1554 bp), were also simultaneously transformed into the protoplasts of strain ZS-1 (for single disruption of *CmOxdc1*) and Δ*CmOxdc3*-7 (for double disruption of *CmOxdc1* and *CmOxdc3*) under the mediation of PEG [16]. The resulting protoplasts were regenerated on TB3 medium at 20 °C and the emerging fungal colonies were individually picked out and transferred to PDA plates followed by incubation at 20 °C and selection of the disruption mutants. Finally, four single disruption mutants (Δ*CmOxdc3*-2, Δ*CmOxdc3*-7, Δ*CmOxdc1*-1, Δ*CmOxdc1*-25) and two double disruption mutants (Δ*CmOxdc1*&*3*-13, Δ*CmOxdc1*&*3*-45) were obtained.

The disrupted *CmOxdc3* in the mutant Δ*CmOxdc3*-7 was complemented using the strategy shown in Figure S2. The 5′-flank region, together with the coding sequence of *CmOxdc3*, excluding the stop codon (2701 bp) and the enhanced green fluorescent protein gene (eGFP, 745 bp) from the plasmid pCHEG (Drs. Guogeng Yang and Daohong Jiang, unpublished), were separately amplified. They were ligated and inserted into the plasmid pSKTN [35] with the aid of *Kp*n I and *Hin*d III to generate the intermediate plasmid pSKTN-Oxdc3-eGFP. Then, the hygromycin resistance gene (*HYG*) from pBluscrikp II KS1004 [16] was inserted into pSKTN-Oxdc3-eGFP at *Xb*a I to generate the final plasmid (pSKTH-*Oxdc3*-eGFP), which was transformed into the protoplasts of Δ*CmOxdc3*-7 mediated by PEG [16], followed by protoplast regeneration on TB3 and selection of the complementary mutants. Finally, a complementary mutant Δ*CmOxdc3*-7C was obtained.

The transformants were screened on PDA amended with hygromycin B (50 µg mL^−1^) or G418 (25 µg mL^−1^) three times and verified by PCR (Figure S1B) with the primers in Table S1 and/or by Southern blotting.

### 2.7. Determination of Sensitivity to OA

The mutants (Δ*CmOxdc1*-1, Δ*CmOxdc1*-25, Δ*CmOxdc3*-2, Δ*CmOxdc3*-7, Δ*CmOxdc3*-7C, Δ*CmOxdc1*&*3*-13, Δ*CmOxdc1*&*3*-45) and the wild type (ZS-1) were separately incubated for 30 days (20 °C) on PDA amended with OA at 0 (control), 8, 16 or 24 mM in Petri dishes. There were three dishes (replicates) for each strain in the control and each OA treatment, and the entire experiment was repeated three times. Diameter of the colony in each dish was measured at 10-day and 30-day post inoculation (dpi). Sensitivity of a strain to OA at a given concentration was calculated based on the colony diameters of that strain in the control (0 mM OA) and each OA treatment [35].

### 2.8. Assaying OA Degradation and Mycoparasitic Activities

The mutants and the wild type (ZS-1) mentioned above were separately inoculated in 150 mL flasks, each containing 50 mL PDB amended with OA to the final concentrations of 0 (control), 12 or 24 mM. Aliquots of the conidial suspension (1 × 10^7^ conidia mL^−1^) of each mutant or ZS-1 were inoculated in the media, 100 μL in each flask, 3 flasks for each mutant and ZS-1. The flasks were shake-incubated at 20 °C and 150 rpm for 12 days. The cultures were separately filtered, and the cultural filtrates were loaded in high performance liquid chromatography (HPLC) to determine the OA concentration [47]. The oxalate degradation rate was calculated using the method described by Zeng et al. [16]. There were three replicates for each treatment, and the entire experiment was conducted three times. Mycoparasitic activity of the wild type and mutants of *C. minitans* to the hyphae and sclerotia of *S. sclerotiorum* was performed using the method described by Lou et al. [35]. Infection of the hyphae was evaluated by the aggressiveness of a mutant (or ZS-1) in the invasion of the colonies of *S. sclerotiorum* in the dual cultures of that mutant (or ZS-1) and *S. sclerotiorum*, and infection of sclerotia by a mutant (or ZS-1) was evaluated by sclerotial rot index [35]. This experiment was also repeated three times.

### 2.9. Confocal Microscopy

To visualize the localization of CmOxDC3, the complementary mutant Δ*CmOxdc3*-7C with the transformed with the eGFP gene (eGFP = enhanced green fluorescent protein) and the eGFP control strain ZS-1-E1 were inoculated in PDB (50 mL per flask) with 1 × 10^7^ conidia in each flask, and the cultures were shake-incubated at 20 °C and 150 rpm for 3 days. Then, OA was added to the flasks to the final concentrations of 0 (control), 8 mM, 16 mM or 24 mM, and the cultures were further incubated for 1 h. The hyphae were collected from each flask and observed under Leica SP8 Inverted Confocal Microscope (Germany).

### 2.10. Statistical Analysis

One-way analysis of variance (ANOVA) in the GraphPad Prism 8.0.2 (https://www.graphpad.com/; accessed on 6 July 2020) was used to determine significant differences among mutants and the wild type (ZS-1) of *C. minitans* in OA degradation rates, sclerotial rot index and relative gene expression levels. Treatment means were separated using Duncan’s multiple range test or Student’s *t* test. 

## 3. Results

### 3.1. Identification and Characteristics of CmOxdc3

**Figure 1:**
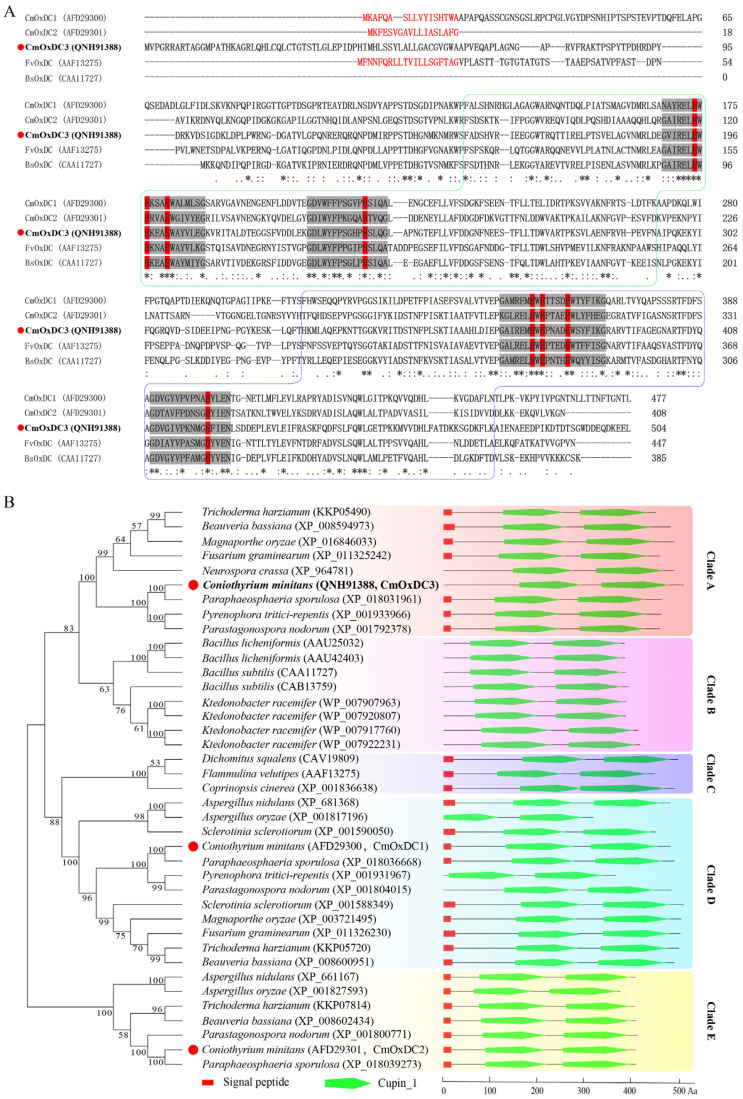
Multiple sequence alignment of oxalate decarboxylases (OxDCs) showing the evolutionary status of CmOxDC3. (**A**) Multiple amino acid sequences of OxDCs. Red letters indicate the region for the secretion signal peptides; Two dashed line boxes represent the cupin domains; Grey areas show the conserved motifs in the Cupin domains; Red areas indicate amino acid residues for Mn^2+^-binding sites; *, the same amino acid residues; chemically-similar amino acid residues; (**B**) Phylogenetic tree for 39 OxDCs. Red dots for CmOxDC1 to 3. The bootstrap values from 1000 replications are given at the branches of the tree. The domains and signal peptides were predicted by SMART and SignalP 5.0, and were displayed on the right of the tree.

The amino acid sequences encoded by *CmOxdc1* (GenBank Acc. No. JF718548) and *CmOxdc2* (GenBank Acc. No. JF718549) were used as queries to search the homologs in the genome of *C. minitans* ZS-1 (GenBank Acc. No. VFEO01000000). A homologous gene was finally found, and the coding region of that gene was 1565 bp long with one intron (50 bp long) and two extrons (333 and 1182 bp long). A polypeptide with 504 amino acids (aa) encoded by the homologous gene was predicted, and it shared 35.76% and 31.56% identity to the polypeptides CmOxDC1 and CmOxDC2 encoded by *CmOxdc1* and *CmOxdc2*, respectively. Moreover, the 504-aa polypeptide harbored a bicupin structure similar to that in CmOxDC1 and CmOxDC2 (Figure 1A). However, different from CmOxDC1 and CmOxDC2, the 504-aa polypeptide did not have a signal peptide. Therefore, the homologous gene was probably a novel oxalate decarboxylase gene, herein designated as *CmOxdc3* (GenBank Acc. No. MN688991). SWISS-MODEL (https://swissmodel.expasy.org/; accessed on 5 March 2022) analysis showed that the predicted structure of CmOxDC3 shared 55.59% identity to that of oxalate decarboxylase in *Bacillus subtilis* (Figure S3). Phylogenetic analysis showed that the 39 OxDCs were grouped into five clades (A to E), CmOxDC3 belonged to Clade A, whereas CmOxDC1 and CmOxDC2 belonged to Clade D and Clade E, respectively (Figure 1B).

### 3.2. Expression Pattern and Subcellular Localization of CmOxdc3

**Figure 2:**
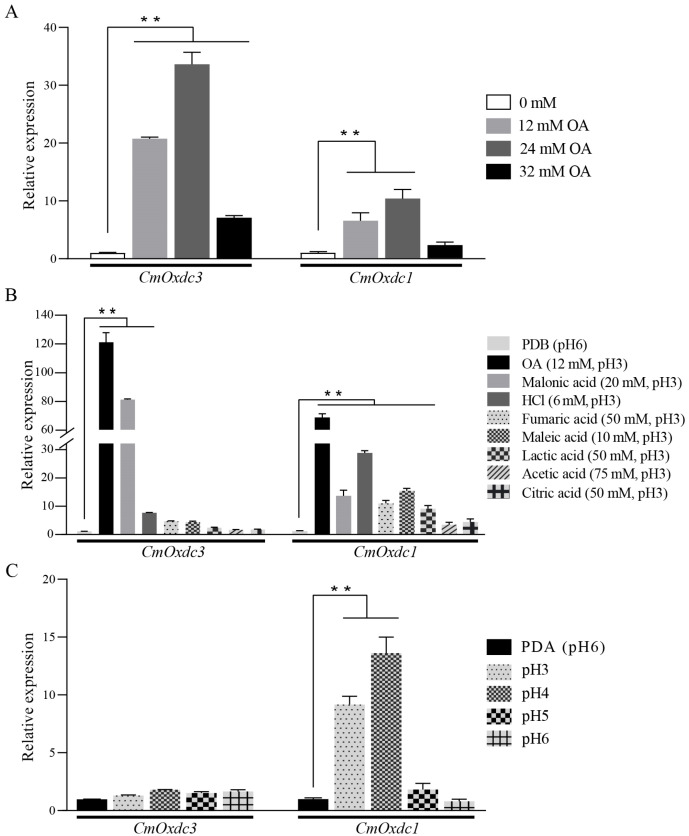
The transcript pattern of *CmOxdc3* and *CmOxdc1* in response to oxalic acid (**A**) and different acids adjusted to pH 3 (**B**) as well as under different ambient pH conditions (**C**). *Cmactin* was used as a reference. ** *p* < 0.01.

The results of RT-qPCR indicated that the wild type ZS-1 of *C. minitans* had a distinct expression pattern of *CmOxdc3* in response to OA and other acids as well as to ambient pH (Figure 2). In the OA assay, expression of *CmOxdc3* was significantly (*p* < 0.01) up-regulated in the presence of OA, compared to the control treatment, the OA treatments (12, 24 and 32 mM) had the average relative expression values (REVs) of *CmOxdc3* being increased by 21, 34 and 7 folds, respectively (Figure 2A). *CmOxdc1* showed a similar expression pattern, the average REVs under the OA treatments were increased by 2 to 10 folds, compared to the control treatment.

Besides OA, malonic acid and HCl could also trigger a significant (*p* < 0.01) up-regulated expression of *CmOxdc3* with the average REVs at 81 and 8, respectively, in the presence of the two acids (Figure 2B). In contrast, the remaining five acids (fumaric acid, maleic acid, lactic acid, acetic acid and citric acid) yielded the average REVs of *CmOxdc3* lower than 4.5, which did not significantly (*p* > 0.05) differ from that in the control treatment. This expression pattern appeared different from that of *CmOxdc1*, which was significantly (*p* < 0.01) up-regulated by OA as well as by malonic acid, HCl, fumaric acid, maleic acid and lactic acid. The average REVs in these six acids ranged from 9 to 68.

In the pH assay, expression of *CmOxdc3* did not have a significant (*p* > 0.05) change under pH 3 to 6. Results also showed that expression of *CmOxdc1* was significantly (*p* < 0.01) increased by 9 and 13 folds under pH 3 and 4, respectively; however, it was not up-regulated under pH 5 and 6 (Figure 2C).

**Figure 3:**
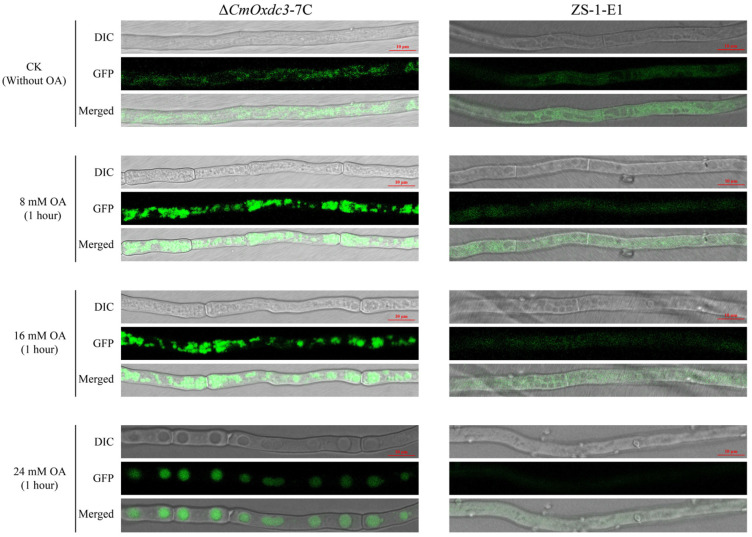
Identification of the subcellular localization of CmOxDC3 (green fluorescent color) in hyphal cells of *Coniothyrium minitans*. Strain ZS-1-E1 constitutively expressing eGFP was used as the control.

To understand the subcellular localization of the polypeptides (CmOxDC3) encoded by *CmOxdc3*, we constructed the complementary mutant Δ*CmOxdc3*-7C, which contained the coding sequence (without stop codon) of *CmOxdc3* and the enhanced green fluorescent protein (eGFP)-coding gene under the native promoter of *CmOxdc3*. Microscopic observation showed that the CmOxDC3-eGFP was visible in the cytoplasm of the transformed hyphae in the absence of OA. With the increase of oxalic acid concentration (from 8 mM to 24 mM), CmOxDC3-eGFP signal was greatly enhanced and gradually gathered. After 1-h 24 mM OA treatment, CmOxDC3-eGFP was obviously gathered to vacuoles in the cytoplasm of the transformed hyphal cells (Figure 3). However, the control eGFP signal was diffusely distribution in the cytoplasm of the control strain (ZS-1-E1) hyphae with or without OA treatment. 

### 3.3. Effect of Disruption of CmOxdc3 on Sensitivity to OA

**Figure 4:**
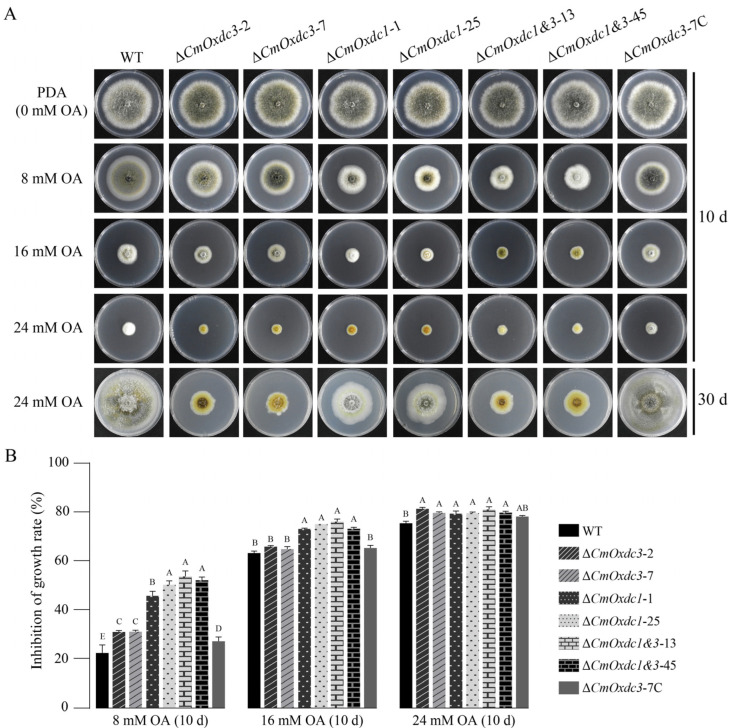
Effect of disruption of *CmOxdc3* and/or *CmOxdc1* in *C. minitans* on its sensitivity to oxalic acid (OA). (**A**) Colony morphology of the disruption mutants and the wild type (WT) on PDA amended with OA; (**B**) Histogram showing mycelial growth-inhibition rates of the mutants and WT in response to OA. Means ± S.D. (*n* = 3) labeled with the same letters for each concentration of OA indicate no significant difference (*p* > 0.01) according to Duncan’s multiple range test.

To explore the specific function of *CmOxdc3*, the gene deletion mutants Δ*CmOxdc3*-2 and Δ*CmOxdc3*-7 were generated using the split-marker strategy (Figure S1A). In addition, the complementary mutant *CmOxdc3*-7C and the double disruption mutants Δ*CmOxdc1*&*3*-13, Δ*CmOxdc1*&*3*-45 were also obtained. Disruption of *CmOxdc3*, as well as *CmOxdc1* and complementation of *CmOxdc3*, were confirmed by PCR and/or Southern blotting (Figure S4). The wild type (ZS-1) and the mutants of *CmOxdc3* as well as the mutants of *CmOxdc1* (Δ*CmOxdc1*-1, Δ*CmOxdc1*-25) were compared for sensitivity to OA on PDA amended with OA at 8, 16 or 24 mM (PDA-OA 8, PDA-OA 16, PDA-OA 24, respectively). The results showed that after incubation at 20 °C for 10 days, both the wild type and all the mutants formed large colonies on PDA alone (control), however, they formed small colonies on PDA amended with OA at 8, 16 and 24 mM (Figure 4A). Therefore, OA can inhibit mycelial growth of the tested strains of *C. minitans*. The results also showed that the colony size varied with the strains of *C. minitans* on PDA-OA 8, 16 and 24. On PDA-OA 8, Δ*CmOxdc3*-2 and Δ*CmOxdc3*-7 had a significantly (*p* < 0.05) higher mycelial growth-inhibition (MGI) rate (31%) than the wild type (22%) and *CmOxdc3*-7C (27%), however, the value was significantly (*p* < 0.05) lower than those (46% to 54%) for Δ*CmOxdc1*-1, Δ*CmOxdc1*-25, Δ*CmOxdc1*&*3*-13, Δ*CmOxdc1*&*3*-45 (Figure 4B). This result suggests that disruption of *CmOxdc3* causes less sensitivity to OA at 8 mM than disruption of *CmOxdc1*.

On PDA-OA 16, Δ*CmOxdc3*-2 and Δ*CmOxdc3*-7 had a similar MGI rate (65%) to that of the wild type (63%) and Δ*CmOxdc3*-7C (65%); however, the MGI values for these four mutants were significantly lower (*p* < 0.05) than those (73% to 76%) for Δ*CmOxdc1*-1, Δ*CmOxdc1*-25, Δ*CmOxdc1*&*3*-13 and Δ*CmOxdc1*&*3*-45 (Figure 4A). This result still suggests that disruption of *CmOxdc3* causes less sensitivity to OA at 16 mM than disruption of *CmOxdc1*.

On PDA-OA 24, all the mutants (Δ*CmOxdc3*-2, Δ*CmOxdc3*-7, Δ*CmOxdc3*-7C, Δ*CmOxdc1*-1, Δ*CmOxdc1*-25, Δ*CmOxdc1*&*3*-13, Δ*CmOxdc1*&*3*-45) and the wild type formed tiny colonies with the MGI rates ranging from 75% to 81% after incubation for 10 days (20 °C). The six disruption mutants (Δ*CmOxdc3*-2, Δ*CmOxdc3*-7, Δ*CmOxdc1*-1, Δ*CmOxdc1*-25, Δ*CmOxdc1*&*3*-13, Δ*CmOxdc1*&*3*-45) had higher MGI rates (79% to 81%) than the wild type (75%) and the complementary mutant Δ*CmOxdc3*-7C (78%) (Figure 4B). However, after incubation for 30 days, a dramatic difference in the size of the colonies was observed among the tested mutants and the wild type (Figure 4A, the bottom row of petri dishes). While the wild type and the complementary mutant Δ*CmOxdc3*-7C colonized the entire dishes, with the average colony diameter reaching up to 9 cm, followed by the *CmOxdc1*-disruption mutants (Δ*CmOxdc1*-1, Δ*CmOxdc1*-25), which had the average colony diameter at 7 cm, the *CmOxdc3*-disruption mutants (Δ*CmOxdc3*-2, Δ*CmOxdc3*-7) and the double disruption mutants (Δ*CmOxdc1*&*3*-13, Δ*CmOxdc1*&*3*-45) formed small colonies with the average colony diameter at 3 cm. This result suggests that disruption of *CmOxdc3* causes more sensitivity to OA at 24 mM than disruption of *CmOxdc1*.

### 3.4. Effect of Disruption of CmOxdc3 on OA Degradation

**Figure 5:**
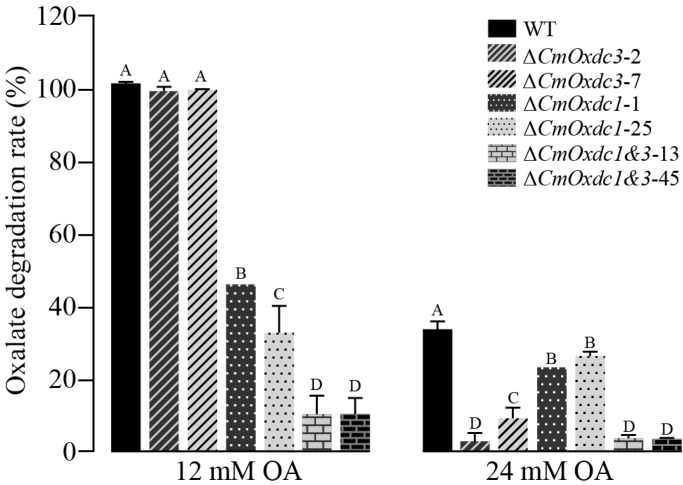
Histogram showing rates of degradation of oxalic acid (OA) by the wild type (WT) and mutants of *C. minitans*. Means ± S.D (*n* = 3) labeled with the same letters for each concentration of OA indicate no significant difference (*p* > 0.01).

After shake-incubation (20 °C, 150 rpm) for 12 d, both the wild type and all of the mutants grew in PDB supplemented with OA, which was degraded, ranging from 3% to 100%. In PDB supplemented with 12 mM OA, both the wild type and the *CmOxdc3*-disruption mutants (Δ*CmOxdc3*-2, Δ*CmOxdc3*-7) almost degraded all OA, whereas the *CmOxdc1*-disruption mutants (Δ*CmOxdc1*-1, Δ*CmOxdc1*-25) and the double disruption mutants (Δ*CmOxdc1*&*3*-13, Δ*CmOxdc1*&*3*-45) degraded a small proportion of OA (33% to 46%) (Figure 5). It appears that *CmOxdc1* plays a major role in degradation of 12 mM OA. In PDB supplemented with 24 mM OA, the rates of OA degradation by the tested mutants and ZS-1 were greatly decreased to 3–33%. Relatively, the wild type had the highest OA-degradation rate (33%), followed by the *CmOxdc1*-disruption mutants (Δ*CmOxdc1*-1, Δ*CmOxdc1*-25) with the average OA-degradation rate of 25%, the *CmOxdc3*-disruption mutants (Δ*CmOxdc3*-2, Δ*CmOxdc3*-7) and the double disruption mutants (Δ*CmOxdc1*&*3*-13, Δ*CmOxdc1*&*3*-45) had the lowest OA-degradation rates (<9%). This result suggests that *CmOxdc3* plays an important role in the degradation of 24 mM OA.

### 3.5. Effect of Disruption of CmOxdc3 on Mycoparasitism on the Host Hyphae

**Figure 6:**
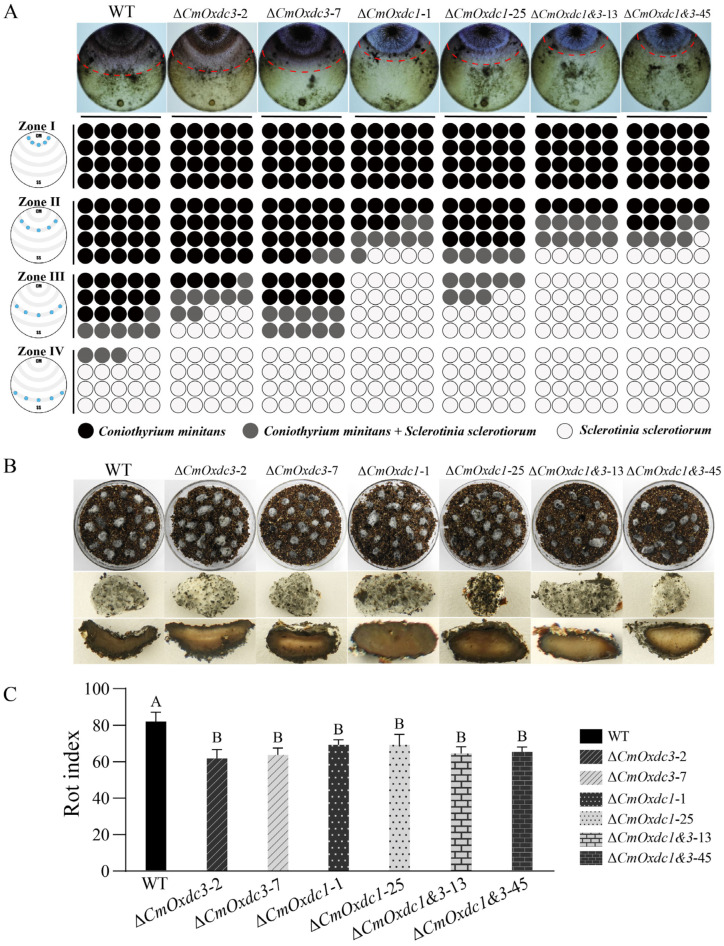
Mycoparasitism of the mutants and the wild type of *C. minitans* on hyphae and sclerotia of *S. sclerotiorum* (20 °C, 30 d). (**A**) Dual cultures between *C. minitans* and *S. sclerotiorum*. A schematic diagram showing the number of agar disks that gave rise to either *C. minitans*, *S. sclerotiorum* or both. Each circle represents a colony developed from a mycelial agar disk sampled from Zones I, II, III or IV in the dual cultures (see the schematic diagram on the left). Red dash lines on the dual-culture plates roughly differentiate the yellow areas from the blue areas. Yellow means low pH due to oxalic acid (OA) secreted by *S. sclerotiorum* and blue represents high pH likely due to OA degradation by *C. minitans*. (**B**) Petri dishes on the top line with the sclerotia of *S. sclerotiorum* infected by different strains of *C. minitans*; The sclerotia in the middle line indicate the typical symptoms of the *C. minitans*-infected sclerotia; and Sections of the *C. minitans*-infected sclerotia on the bottom line. (**C**) Histogram showing sclerotial rot indices caused by different strains of *C. minitans*. Means ± SD (*n* = 3) labeled with the same letters indicate no significant difference (*p* > 0.01).

The dual-cultural assay was used to determine mycoparasitic infection of the wild type and the mutants of *C. minitans* on the hyphae of *S. sclerotiorum* (20 °C, 30 days). The assay was established on PDA amended with bromophenol blue (a pH indicator) to monitor the production of OA by *S. sclerotiorum* as shown by the change of the medium color from blue to yellow (Figure 6A). The result showed that the wild type and all the mutants could invade the colonies of *S. sclerotiorum* in the dual cultures, thereby destroying the hyphae of *S. sclerotiorum*. However, the mutants differed from the wild type in invasion aggressiveness, while the wild type invaded the colonies of *S. sclerotiorum* from Zone I (close to the inoculation point of *C. minitans*) to Zone IV (close to the inoculation point of *S. sclerotiorum*), the mutants Δ*CmOxdc3*-2 and Δ*CmOxdc3*-7, as well as Δ*CmOxdc1*-1, invaded the colonies of *S. sclerotiorum* from Zone I to Zone III, and the remaining mutants Δ*CmOxdc1*-25, Δ*CmOxdc1*&*3*-13 and Δ*CmOxdc1*&*3*-45 invaded the colonies of *S. sclerotiorum* from Zone I and Zone II. This result suggests that disruption of *CmOxdc3*, as well as *CmOxdc1*, can delay invasion into the colonies of *S. sclerotiorum* possibly due to reduced capability for eliminating the toxicity of OA from *S. sclerotiorum*.

The sclerotial assay was used to determine the mycoparasitic infection of the wild type and the mutants of *C. minitans* on the sclerotia of *S. sclerotiorum* (Figure 6B). The result showed that after incubation at 20 °C for 30 days, the wild type and the mutants could infect the sclerotia of *S. sclerotiorum*, causing blackening and rot of the sclerotia. The average sclerotial rot index (0 to 100) was as high as 82 for the wild type, however, the value was significantly (*p* < 0.05) reduced to 62–69 for the mutants (Figure 6C). Therefore, disruption of *CmOxdc3*, as well as *CmOxdc1*, can reduce the capability of *C. minitans* to infect and destroy the sclerotia of *S. sclerotiorum*.

## 4. Discussion

Oxalic acid (OA) plays various roles for the OA-producing fungal pathogens to infect plants [5,6], and microbial degradation of OA is a valuable research topic regarding the initiation of novel control strategies against the OA-producing fungi [6,11,15,16,17,18,19,20]. *C. minitans* is a promising biological control agent of *S. sclerotiorum* [28,29,30,31,32]. It is of great significance to study the role and molecular mechanisms for OA degradation by *C. minitans*. In this study, we cloned a novel OA degradation-associated gene (*CmOxdc3*) from *C. minitans*, which may work in a synergistic manner with *CmOxdc1*. *CmOxdc3* and *CmOxdc1* share the conserved bicupin structure, and the expression of both genes can be triggered by OA. However, *CmOxdc3* differs from *CmOxdc1* in two aspects. First, *CmOxdc3* does not have a signal peptide-coding region, whereas *CmOxdc1* has a signal peptide-coding region. As a consequence, CmOxDC3 is located in hyphal cytoplasm as observed in this study. In addition, *CmOxdc3* had no detectable response to fumaric acid, maleic acid, lactic acid, acetic acid and citric acid, as well as the ambient acidic pH signaling, whereas *CmOxdc1* could be triggered for expression by these acids and acidic pH signaling.

In the process of evolution, microorganisms usually retain multiple genes to cope with the same biochemical processes, such as microbial degradation of OA. This polygenic phenomenon usually has a positive impact on the survival of microorganisms. Previous studies showed that the presence of multiple oxalate decarboxylases (OxDCs) is very common in fungi and bacteria [16], this phenomenon implies that OA degradation by fungi or bacteria is a complex biochemical process. Zeng et al. (2014) cloned two oxalate decarboxylase genes (*CmOxdc1* and *CmOxdc2*) from *C. minitans* and proved that *CmOxdc1* is important for *C. minitans* to degrade OA [16]. Two oxalate decarboxylase genes (*Ss-odc1* and *Ss-odc2*) have been reported in *S. sclerotiorum*, and only *Ss-odc2* is responsible for degradation of OA [11]. In this study, we found a novel oxalate decarboxylase gene (*CmOxdc3*) in *C. minitans*, which had a distinct structure and expression pattern from *CmOxdc1* and *CmOxdc2*. This study found that *CmOxdc3* also plays a certain role in degradation of OA. This result, together with our previous observation about the role of *CmOxdc1*, suggests that *CmOxdc1* and *CmOxdc3* might be responsible for OA degradation in *C. minitans*.

Previous studies indicated that fungal oxalate decarboxylases have diverse subcellular localization [23,24,25,26]. This study showed that CmOxDC3 was localized in the cytoplasm of hyphal cells; it moved to vacuoles in response to extracellular OA. Vacuoles in fungi are similar to lysosomes in mammalian cells. They are acidified organelles, which have the function of maintaining cell pH homeostasis, storage and recycling of nutrients, degradation of macromolecules and membrane transport [48,49]. In *C. minitans*, disruption of the vacuolar morphogenesis-related *CmVps39* affected osmotic adaptation, pH homeostasis and cell wall integrity [50]. Therefore, vacuoles may be the main site for intracellular degradation of OA. Intracellular localization of CmOxDC3 suggests that it may differ from CmOxDC1 in degradation of OA, whereas CmOxDC1 has a signal peptide region, and CmOxDC1 can be transported outside of the hyphal cells (Method S1, Figure S5) to degrade extracellular OA.

Based on the results so far achieved, we proposed a model for the synergistic degradation of OA by CmOxDC1 and CmOxDC3 in the interaction between *C. minitans* and *S. sclerotiorum*. *S. sclerotiorum* produces OA, which is transported outside. Partial OA may enter into the hyphal cells of *C. minitans* (possibly gathers in vacuoles) by an unknown way. Meanwhile, *C. minitans* senses OA via unknown receptors, and the resulting signals are then transmitted to nuclei, thereby activating the transcription of *CmOxdc1*/*3*. CmOxDC3 degrades the intracellular OA, whereas CmOxDC1, with the signal peptide, is secreted to the extracellular matrix to degrade extracellular OA.

In summary, this study cloned a novel oxalate decarboxylase gene (*CmOxdc3*) in *C. minitans*. It did not harbor the signal peptide region, it was found to locate inside the hyphal cells, gathering in vacuoles. Transcription of *CmOxdc3* was triggered by OA, malonic acid and HCl, but failed to respond to ambient acidic pH from 3 to 6. Disruption of *CmOxdc3* increased sensitivity of *C. minitans* to OA as well as reduced the efficiency of *C. minitans* to degrade OA and to infect *S. sclerotiorum*. Therefore, *CmOxdc3* plays a different role from that of *CmOxdc1*, and both *CmOxdc1* and *CmOxdc3* may work in a synergistic manner to eliminate the toxicity of OA.

## Data Availability

Not applicable.

## Supplementary Materials

The following supporting information can be downloaded at: https://www.mdpi.com/article/10.3390/jof8121304/s1, Figure S1: Schematic diagrams showing the strategy for disruption of the oxalate decarboxylase genes in *C. minitans* (A) and the primers for PCR confirmation of the gene disruption (B); Figure S2: A diagram showing the strategy for the construction of the *CmOxdc3* complementation plasmid; Figure S3: The predicted crystal structure of CmOxDC3 and BsOxDC (SWISS-MODEL Template Library ID: 5vg3.1.A) showing their similarity in topology; Figure S4: Confirmation of disruption and complementation of the oxalate decarboxylase genes *CmOxdc3* and *CmOxdc1* in *C. minitans*. (A) Agarose electrophoregram showing DNA bands PCR amplified from the wild type (WT) and the mutants; (B) A schematic diagram showing the strategy for Southern blotting confirmation of disruption of *CmOxdc3*; (C) A Southern blotting image for confirmation of disruption of *CmOxdc3*; Figure S5: Investigation of secretory property of CmOxDC1 signal peptides by a yeast signal trap system; Method S1: Yeast signal sequence trap system [51]; Table S1: Primers used in this study [16]; Table S2: Sequences of 39 OXDCs to construct phylogenetic tree.
