# Supplementary material for: Cloning and Molecular Characterization of CmOxdc3 Coding for Oxalate Decarboxylase in the Mycoparasite Coniothyrium minitans
Source: J Fungi (Basel). 2022 Dec 16;8(12):1304. doi: 10.3390/jof8121304 (PMC9785797; doi:10.3390/jof8121304)
Supplement: Supplementary file 1 [file jof-08-01304-s001.zip › jof-2050140-supplementary.pdf]

Supplementary Material

Cloning and Molecular Characterization of *CmOxdc3* Coding for Oxalate Decarboxylase in the Mycoparasite *Coniothyrium minitans*

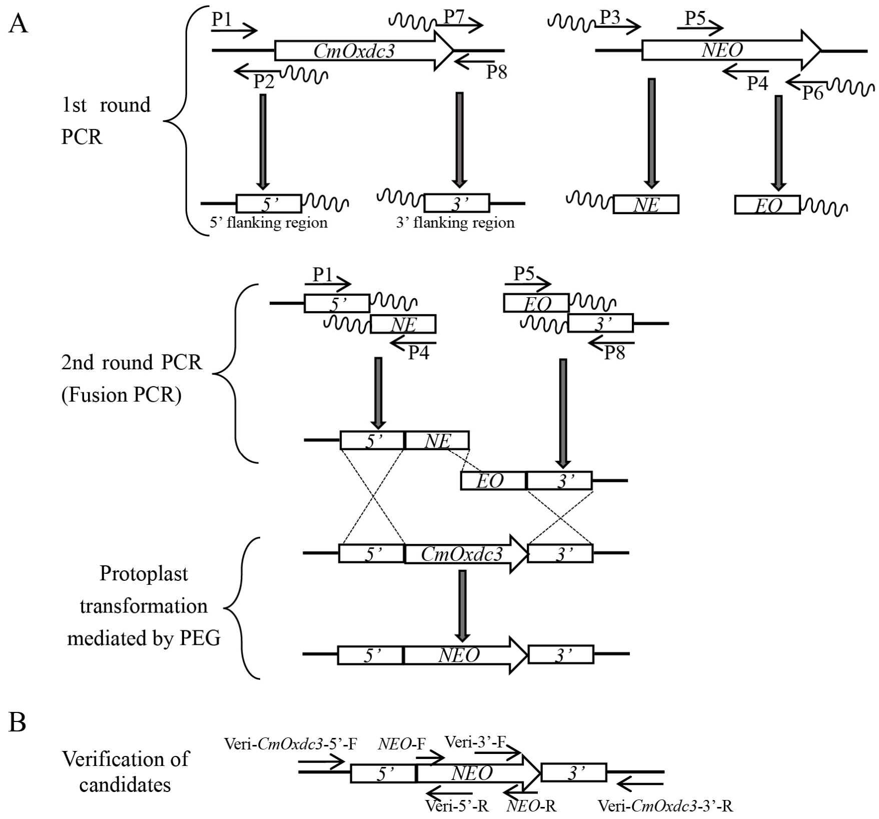

Note: For *CmOxdc1*, the fragments *NEO*, *NE*, *EO* and the primers *NEO*-F&*NEO*-R were changed to *HYG*, *HY*, *YG* and *HYG*-F&*HYG*-R, respectively.

Figure S1. Schematic diagrams showing the strategy for disruption of the oxalate decarboxylase genes in *C. minitans* (**A**) and the primers for PCR confirmation of the gene disruption (**B**).

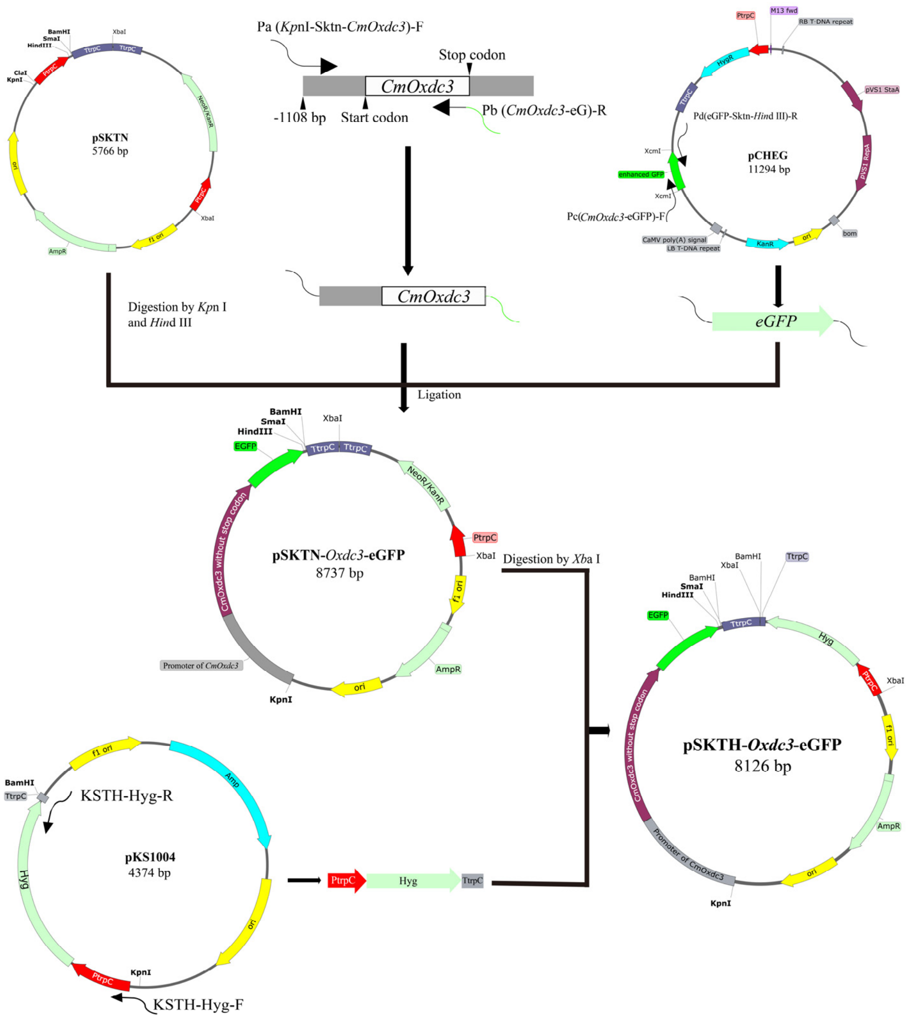

Figure S2. A diagram showing the strategy for the construction of the *CmOxdc3* complementation plasmid.

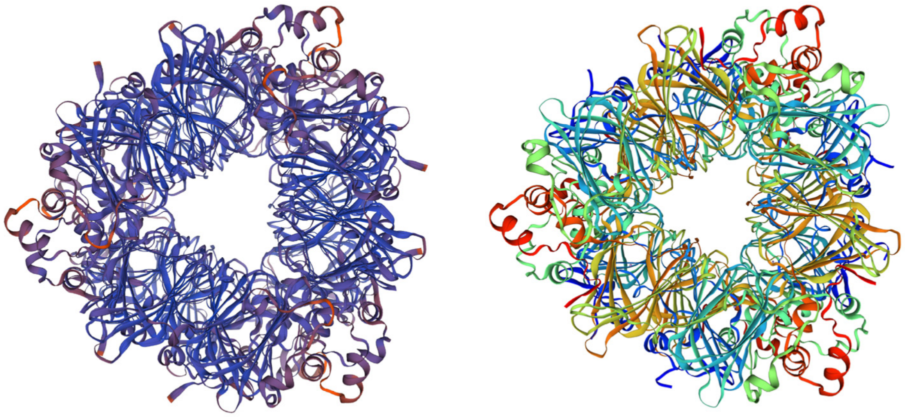

Figure S3. The predicted crystal structure of CmOxDC3 and BsOxDC (SWISS-MODEL Template Library ID: 5vg3.1.A) showing their similarity in topology.

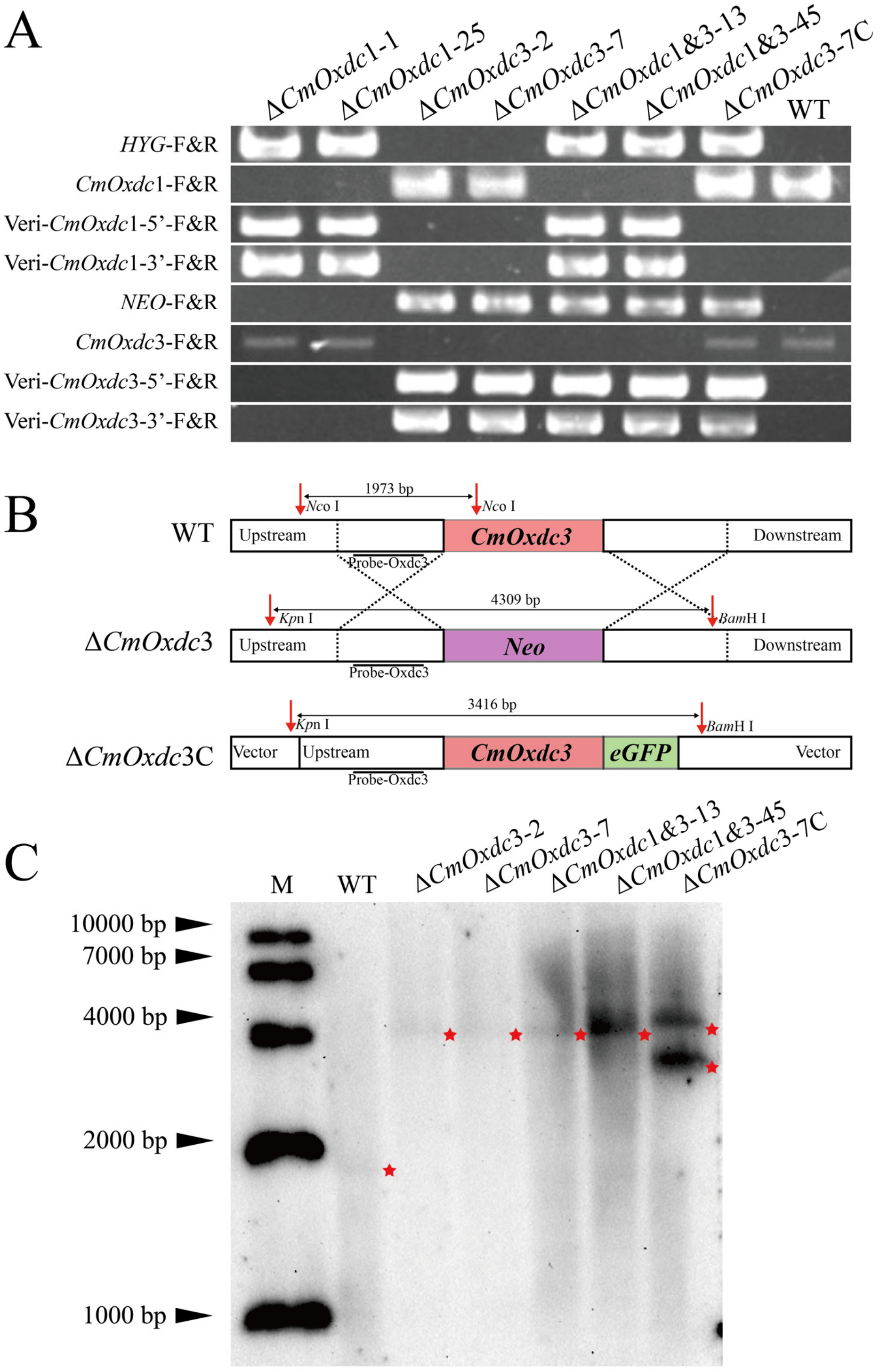

Figure S4. Confirmation of disruption and complementation of the oxalate decarboxylase genes *CmOxdc3* and *CmOxdc1* in *C. minitans*. (**A**) Agarose electrophoregram showing DNA bands PCR amplified from the wild type (WT) and the mutants; (**B**) A schematic diagram showing the strategy for Southern blotting confirmation of disruption of *CmOxdc3*; (**C**) A Southern blotting image for confirmation of disruption of *CmOxdc3*.

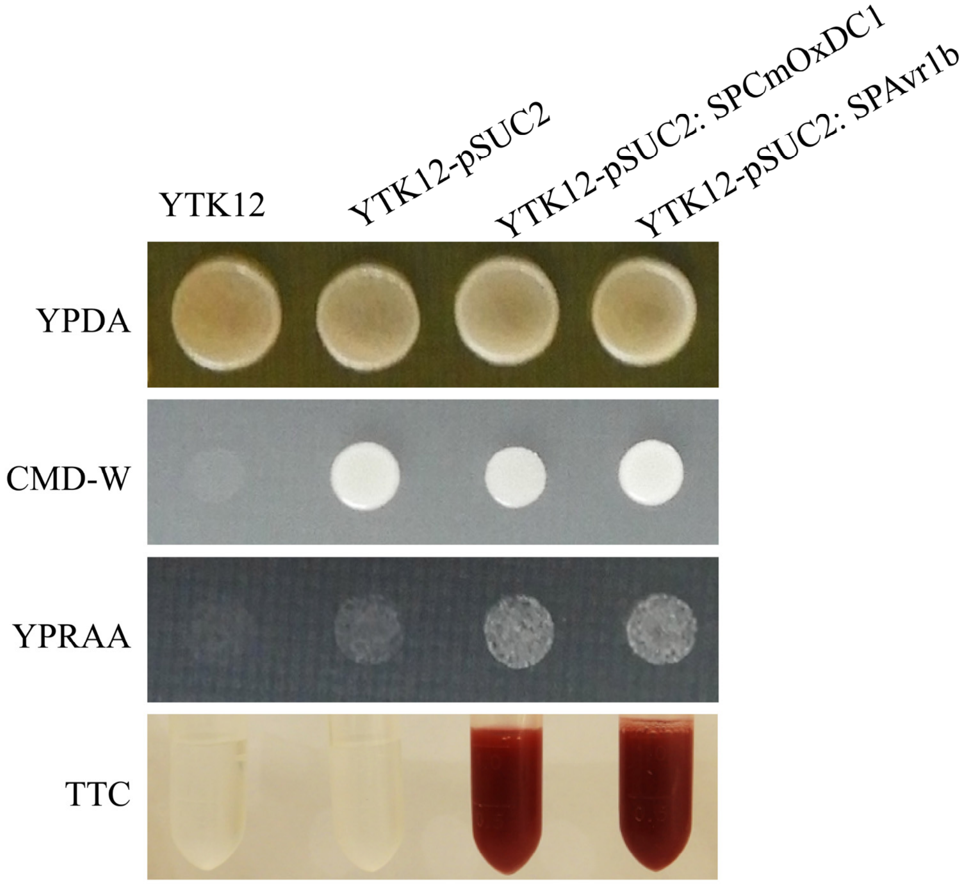

Figure S5. Investigation of secretory property of CmOxDC1 signal peptides by a yeast signal trap system.

Method S1. Yeast signal sequence trap system. To verify the predicted signal peptide of CmOxDC1 is functional or not, the yeast signal sequence trap assay was conducted as previously described [51]. The coding region of the predicted signal peptide of CmOxDC1 (17 amino acids) was amplified, and ligated into the pSUC2 vector, which contained a secretion-defective invertase gene and was digested by *Eco*R I and *Xh*o I. The generated plasmid, pSUC2:SPCmOxDC1, was transformed into the yeast strain YTK12 using the lithium acetate method to yield YTK12-pSUC2:SPCmOxDC1. The positive clones with correct gene sequencing were planted on YPRAA plates for detecting invertase secretion, and further checked by the insoluble red triphenylformazan reduction from 2, 3, 5-triphenyltetrazolium chloride (TTC). The empty pSUC2 vector and the pSUC2:SPAvr1b vector were transformed into yeast strain YTK12, and the resulting strains were designated negative and positive control, respectively. All primers used in this assay are listed in Table S1.

Table S1. Primers used in this study

Primer	Sequence (5′ → 3′)	Purpose	Notes
**For fluorescence quantitative PCR (RT-qPCR) analysis**			
qRT-*CmActin*-F	TCGTGACTTGACCGACTACCTC	To determine expression of *CmActin*	[16]
qRT-*CmActin*-R	TTGCCAATGGTGATGACCTGA		
qRT-*CmOxdc1*-F	TCTCATTAACAGCTCCTACCCG	To determine expression of *CmOxdc1*	[16]
qRT-*CmOxdc1*-R	AGAACACAGACCAACTTCCCAT		
qRT-*CmOxdc3*-F	GGCAACTTCAACGAGGAGTC	To determine expression of *CmOxdc3*	-
qRT-*CmOxdc3*-R	GCCAGCATCTTGTGAGTGAA		
**For construction of the genetic transformation vectors and detection of *CmOxdcx***			

CmOxdc3-F<br>*CmOxdc3*-R	ATGGTTCCAGGGAGGCGAGC<br>TCACAACTCTTCCTTGTCCTGCTCG	Amplification of full CmOxdc3 for gene knockout or complementation verification	-
P1(*CmOxdc3*-5′-F)<br>P2(*CmOxdc3*-5′-R)	CAGCAACTGGAGCGTGTACG<br>CCAGGGAAA-TAGCCGTTCACCCATCGCGAAGAC-GCGAATG	Amplification of upstream homologous fragment of CmOxdc3	P1(*CmOxdc3*-5′-F) and P4(Neo-R, Ne split) were used to amplify the fused fragment, *CmOxdc3*-5′-NE.
P3(*CmOxdc3*-Ne5′-F)<br>P4(*Neo*-R, Ne split)	CATTCGCGTCTTCGCGATGGATGCTCCTCTTCGCTATTACGC<br>CAACGCTATGTCCTGATAGCG	Amplification of upstream partial fragment NEO	
P5(*Neo*-F, oR split)<br>P6(*CmOxdc3*-oR3′-R)	CCTGTCATCTCACCTTGCTC<br>CGCGTCGCAATGCGATGGTACGG-CATAGTGCGTGTTTATGC	Amplification of downstream partial fragment NEO	P5(*Neo*-F, oR split) and P8(*CmOxdc3*-3′-R) were used to amplify the fused fragment, EO-*CmOxdc3*-3′.
P7(*CmOxdc3*-3′-F)<br>P8(*CmOxdc3*-3′-R)	GCATAAACACGCACTATGCCGTAC-CATCGCATTGCGACGCG<br>CACTGCTACTGCCGTTGGAG	Amplification of downstream homologous fragment of CmOxdc3	
Veri-*CmOxdc3*-5′-F<br>Veri-*Neo*-5′-R	TGGAGCACTCTCGATGTGAC<br>GTCACATCGAGAGTGCTCCA	PCR validation for upstream homologous recombination of *CmOxdc*3	-
Veri-*Neo*-3′-F<br>Veri-*CmOxdc3*-3′-R	GCACGTAAAGTGCCAAAGGC<br>CACCAGTGGCACTACCATTG	PCR validation for downstream homologous recombination of *CmOxdc*3	-
NEO-F<br>*NEO*-R	ATGATTGAACAAGATGGATTGCACGC<br>TCAGAAGAACTCGTCAAGAAGGC	Amplification of full *NEO* for gene knockout verification	-
Pa(*Kp*n I-Sktn-*CmOxdc3*)-F<br>Pb(*CmOxdc*3-eG)-R	agggaacaaaagctg**ggtacc**TGCAG-CAGCGGTCCATAGTG (*Kp*n I-site in bold)<br>tgctcaccatCAACTCTTCCTTGTCCTGCTCG	Amplification of 5′ flank and coding sequence (without stop codon) of *CmOxdc3*	To construct the complementation vector pSKTH-*Oxdc3*-eGFP
Pc(*CmOxdc3*-*eGFP*)-F<br>Pd(*eGFP*-Sktn-*Hin*d III)-R	ggaagagttgATGGTGAGCAAGGGCGAGG<br>caggaattcgatatc**aagctt-**GTACAGCTCGTCCATGCCGA (*Hin*d III-site in bold)	Amplification of full *eGFP* from the plasmid pCHEG	
SKTH-*HYG*-F<br>SKTH-*HYG*-R	cgcggtggcggccgc**tctaga**CTG-CAGCCCAACTGATATTGAA (*Xb*a I-site in bold)<br>actgaggaatccgct**tctaga**ACTAGTG-GATCCCCCAACTGG *(Xb*a I-site in bold)	Amplification of full *HYG* cassette from the vector pBluscrikp II KS1004	
HYG-F<br>*HYG*-R	ATGAAAAAGCCTGAACTCACCGC<br>CTATTCCTTTGCCCTCGGAC	Amplification of full *HYG* for gene knockout or complementation verification	-
Psb-*Cmoxdc*3-R	CCATCGCGAAGACGCGAATG	Combine with P1(*CmOxdc3*-5′-F) to clone Probe-oxdc3 for Southern blotting	-

CmOxdc1-F	ATGAAGGCCTTCCAAGCATCG	Amplification of full *CmOxdc1* for gene knockout verification	-
CmOxdc1-R	TCACAAAGTGTTCCCTGTGAAG		
P1(*CmOxdc1*-5′-F)	CGTGCCTTAACACTTACGAGG	Amplification of upstream homologous fragment of *CmOxdc1*	P1(*CmOxdc1*-5′-F) and P4(HY5′-R, HY split) were used to amplify the fused fragment, *CmOxdc1*-5′-HY.
P2(*CmOxdc1*-5′-R)	CTCCACTAGCTCCAGCCAAGGAGGA-GATTCCGAACGAAGC		
P3(*CmOxdc1*-HY5′-F)	GCTTCGTTCGGAATCTCCTCCTTGGCTG-GAGCTAGTGGAG	Amplification of upstream partial fragment *HYG*	
P4(HY5′-R, HY split)	GGATGCCTCCGCTCGAAGTA		
P5(HY3′-F, YG split)	CGTTGCAAGACCTGCCTGAA	Amplification of downstream partial fragment *HYG*	P5(HY3′-F, YG split) and P8(*CmOxdc1*-3′-R) were used to amplify the fused fragment, YG-*CmOxdc1*-3′.
P6(*CmOxdc1*-YG3′-R)	CCTTCTCACCCATCTGCCTTC-TATTCCTTTGCCCTCGGAC		
P7(*CmOxdc1*-3′-F)	GTCCGAGGGCAAAGGAA-TAGAAGGCAGATGGGTGAGAAGG	Amplification of downstream homologous fragment of *CmOxdc1*	
P8(*CmOxdc1*-3′-R)	GGTAACGATGGTTCCTAACTGC		
Veri-*CmOxdc1*-5′-F	GTCCGATCTACCATGCTTTGC	PCR validation for upstream homologous recombination of *CmOxdc1*	-
Veri-*HYG*-5′-R	AAGAGTCACACTTCGAGCGC		
Veri-*HYG*-3′-F	GCAATTTCGATGATGCAGCTTGG	PCR validation for downstream homologous recombination of *CmOxdc1*	-
Veri-*CmOxdc1*-3′-R	CTATGCGTTGAAAAACTCTCGTC		
**For verification of the secretory signal peptide of CmOxDC1**			
pSUC2-F	TTTGTTTCCTCGTCATTGTTCTCG	For PCR detection of recombinant vector pSUC2	-
pSUC2-R	CAGCCCTTGTTGGGTGTGAA		
pS-Ox1-*Eco*R1-F	cggaattttaattaa-**gaattc**ATGAAGGCCTTCCAAGCATCG (*Eco*R I-site in bold)	For construction vector pSUC2-SPCmOxDC1	-
pS-Ox1-*Xho*1-R	cactatagggagaacc**ctcgag**AC-CTGAATTTCCACAGGAAGAAGC (*Xho* I-site in bold)		-

Table S2. Sequences of 39 OXDCs to construct phylogenetic tree

Species	Gene ID or symbol	Length of predicted signal peptides
Aspergillus nidulans	XP_681368	22
	XP_661167	17
Aspergillus oryzae	XP_001827593	17
	XP_001817196	None
Bacillus licheniformis	AAU25032	None
	AAU42403	None
Bacillus subtilis	CAA11727, BsOxDC	None
	CAB13759	None
Beauveria bassiana	XP_008594973	22
	XP_008600951	19

	XP_008602434	16
Coniothyrium minitans	AFD29300, CmOxDC1	17
	AFD29301, CmOxDC2	18
	QNH91388, CmOxDC3	None
Coprinopsis cinerea	XP_001836638	19
Dichomitus squalens	CAV19809	20
Flammulina velutipes	AAF13275, FvOxDC	20
Fusarium graminearum	XP_011325242	18
	XP_011326230	22
Ktedonobacter racemifer	WP_007907963	None
	WP_007920807	None
	WP_007917760	None
	WP_007922231	None
Magnaporthe oryzae	XP_016846033	19
	XP_003721495	16
Neurospora crassa	XP_964781	None
Paraphaeosphaeria sporulosa	XP_018036668	17
	XP_018039273	18
	XP_018031961	18
Parastagonospora nodorum	XP_001804015	None
	XP_001800771	18
	XP_001792378	17
Pyrenophora tritici-repentis	XP_001931967	None
	XP_001933966	17
Sclerotinia sclerotiorum	XP_001588349	22
	XP_001590050	22
Trichoderma harzianum	KKP05720	20
	KKP07814	18
	KKP05490	18
